# Bio-electrospraying assessment toward in situ chondrocyte-laden electrospun scaffold fabrication

**DOI:** 10.1177/20417314211069342

**Published:** 2022-01-08

**Authors:** Ângela Semitela, Gonçalo Ramalho, Ana Capitão, Cátia Sousa, Alexandrina F Mendes, Paula AAP Marques, António Completo

**Affiliations:** 1 Centre of Mechanical Technology and Automation (TEMA), Department of Mechanical Engineering, University of Aveiro, Aveiro, Portugal; 2 Centre for Neuroscience and Cell Biology (CNC), University of Coimbra, Coimbra, Portugal

**Keywords:** Cartilage tissue engineering, electrospraying, chondrocyte, needle gauge, operational parameters

## Abstract

Electrospinning has been widely used to fabricate fibrous scaffolds for cartilage tissue engineering, but their small pores severely restrict cell infiltration, resulting in an uneven distribution of cells across the scaffold, particularly in three-dimensional designs. If bio-electrospraying is applied, direct chondrocyte incorporation into the fibers during electrospinning may be a solution. However, before this approach can be effectively employed, it is critical to identify whether chondrocytes are adversely affected. Several electrospraying operating settings were tested to determine their effect on the survival and function of an immortalized human chondrocyte cell line. These chondrocytes survived through an electric field formed by low needle-to-collector distances and low voltage. No differences in chondrocyte viability, morphology, gene expression, or proliferation were found. Preliminary data of the combination of electrospraying and polymer electrospinning disclosed that chondrocyte integration was feasible using an alternated approach. The overall increase in chondrocyte viability over time indicated that the embedded cells retained their proliferative capacity. Besides the cell line, primary chondrocytes were also electrosprayed under the previously optimized operational conditions, revealing the higher sensitivity degree of these cells. Still, their post-electrosprayed viability remained considerably high. The data reported here further suggest that bio-electrospraying under the optimal operational conditions might be a promising alternative to the existent cell seeding techniques, promoting not only cells safe delivery to the scaffold, but also the development of cellularized cartilage tissue constructs.

## Introduction

Tissue engineering (TE) strategies have been actively seeking for an optimal approach for the development of suitable articular cartilage tissue replacements, given that the current treatment options do not constitute a feasible long-term solution.^1^ Considerable efforts have been made to improve scaffolds design—choice of material and fabrication technique, topography, and three-dimensional (3D) anisotropic design—for functional cartilage tissue formation support, as well as effective cell incorporation and subsequent interaction of host cells within the construct.^2,3^ Electrospinning, for instance, has been widely employed for the fabrication of fibrous scaffolds for cartilage TE, not only due to its simplicity and versatility, but also the ECM-mimicking nanofibers produced, known to trigger a suitable chondrocyte response.^4–9^ Still, the pores generated by electrospinning are usually too small to allow effective cell migration into the inner regions of the scaffold, particularly in 3D designs, resulting in poor and time dependent cellular infiltration, and ultimately, in the production of non-functional tissue constructs.^10–13^ In this regard, a logical conclusion would be to directly incorporate the cells into the fibers mesh during scaffolds production in order to fabricate functional and homogeneous tissue constructs, by overcoming the challenges of cell infiltration through small pores by literally surrounding the cells with the fiber matrix as it is produced. Indeed, there are reports of successful development of cell-laden scaffolds by combining fiber electrospinning with cell electrospraying.^14–17^ Cell electrospraying, or bio-electrospraying, a concept first introduced in 2005 by Jayasinghe et al.,^18,19^ enables the deposition of living cells onto specific targets by exposing the cell suspension to an external high intensity electric field. The principle underlying electrospraying involves the application of voltage on a capillary holding the flow of liquid media, resulting in the ejection of a liquid microjet of charged droplets onto an oppositely charged collector. Moreover, when an electric potential difference threshold between the capillary and the collector is achieved, a stable conical liquid meniscus is formed—Taylor cone.^20–23^ Concerning cell electrospraying, the establishment of this stable cone-jet is crucial for the control of the precise cell placement, and it requires certain operational conditions, such as a particular flow rate, surface tension, conductivity, and voltage.^23^ Still, it is necessary to understand how the exposure to the electric field, as well as shear stress of passing through the cell electrospraying apparatus may affect cell viability and function. So far, neuronal cells,^18,24,25^ smooth muscle cells,^26–28^ lymphocytes,^29^ mononuclear cells,^30^ primary cardiac myocytes and endothelial cells,^31,32^ kidney cells,^33^ embryonic stem cells,^34^ to hematopoietic stem cells,^35^ and even for multicellular organisms^36^ have been electrosprayed and survived with no significant influence on a genetic, genomic, and physiological level. Despite the fact that electrospraying experiments have been undertaken on mesenchymal stem cells,^37–41^ whose subsequent chondrogenic differentiation has been examined, no research to the authors’ knowledge have documented electrospraying cells with an unambiguously chondrogenic phenotype. Thus, the purpose of this work is to ascertain the effect of the electrospraying technique and its associated parameters on the survivability and proliferative activity of chondrocytes in order to enable the manufacture of chondrocyte-laden scaffolds for cartilage TE using this technology associated with electrospinning.

## Materials and methods

All experiments were performed in a NANON 01 electrospinning machine (MECC; Fukuoka, Japan), thoroughly cleaned with 70 % (v/v) ethanol beforehand. The remaining used instruments were already sterile or autoclaved before use. Before each experiment, 5 mL of Phosphate-Buffered Saline (PBS; Sigma Aldrich) supplemented with 2.5 µg/mL Amphotericin B (Sigma-Aldrich) was passed through the electrospraying and electrospinning apparatus.

### Electrospraying apparatus

**Figure 1.**
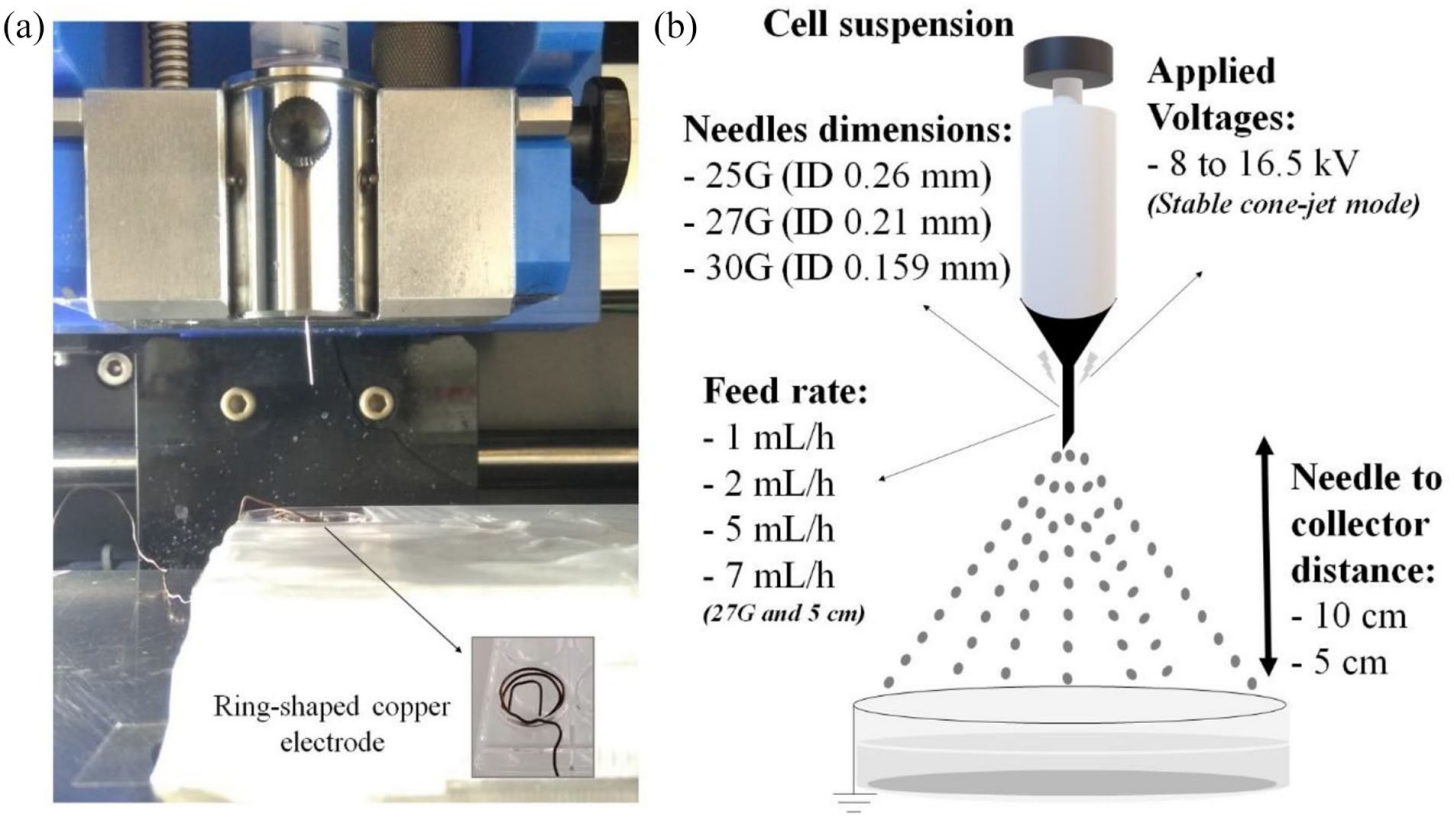
Electrospraying apparatus (a), and illustrative diagram of the electrospraying parameters used for the chondrocyte electrospraying experiments (b).

The experimental set-up is summarized in Figure 1. Stainless-steel needles with varying internal diameters (ID) were connected to a high voltage power supply with the ability to supply up to 30 kV. The needles were attached to cell suspension-containing 5 mL plastic syringes. The samples were collected in culture medium containing-wells of 24-well plates having ring-shaped copper grounded electrodes on its surface (Figure 1(a)).

### Chondrocyte culture

#### Human chondrocyte cell line C28/I2

An immortalized human chondrocyte cell line C28/I2 (kindly provided by Prof. Mary Goldring, Hospital for Special Surgery, New York and Harvard University) was used. Cells were maintained at 37°C in a humidified atmosphere of 5% CO_2_ in air, in Dulbecco’s Modified Eagle Medium (DMEM)/Nutrient Mixture F-12 Ham 1:1 v/v (DMEM: Gibco, Life Technologies; F-12: Sigma-Aldrich) supplemented with 10% (v/v) non-heat-inactivated Fetal Bovine Serum (FBS; Gibco, Life Technologies), 1% (v/v) Penicillin/Streptomycin (P/S; Grisp). Medium refreshments were performed two times a week. Cells were harvested at pre-confluence at passage 26 using trypsin/EDTA solution (0.05%/0.02%, Sigma-Aldrich) for the electrospraying experiments.

#### Primary chondrocytes

Human knee cartilage was collected within 24 h of death from the distal femoral condyles of multi-organ donors at the Bone and Tissue Bank of the University and Hospital Centre of Coimbra (CHUC). Only waste tissue resulting from the preparation of bone tissue for cryopreservation was used. All procedures were approved by the Ethics Committee of CHUC (protocol approval number 8654/DC), which follows the Declaration of Helsinki and Oviedo Convention and the Portuguese legislation for organ donation.

Human chondrocytes were isolated by enzymatic digestion from cartilage samples as previously described.^42^ Briefly, cartilage shavings underwent sequential digestion with Pronase (Roche, Indianapolis, IN, USA) and collagenase A (Roche, Indianapolis, IN, USA) and then suspended in F-12 supplemented with 10% (v/v) non-heat-inactivated FBS and 1% P/S and maintained at 37°C in a humidified atmosphere of 5% CO_2_ in air. Medium refreshments were performed twice a week. Cells were harvested at pre-confluence using trypsin/EDTA solution for the electrospraying experiments.

### Electrospraying optimization

Afterwards, C28/I2 chondrocyte were split into three groups, each with 1 × 10^6^ chondrocytes suspended in 300 µL of culture medium with 0.25 µg/mL Amphotericin B: culture controls (CC), which were maintained in the laminar flow hood at room temperature during the electrospraying process; needle control (NC), where the cell suspensions were subjected to the mechanical stress of passing through the electrospraying apparatus (using a low feed rate); and electrosprayed samples (*E*), where the cell suspensions were pumped through the electrospraying apparatus and exposed to voltage. Several electrospraying parameters were tested (Figure 1(b)): three needle gauges (NG), the gauge of a needle refers to the size of the hole in the needle, the higher the gauge, the smaller the hole (25G—0.26 mm ID, 27G—0.2 mm ID, and 30G—0.159 mm ID, all with 15 mm length), two needle to collector distances (NCD) (5 and 10 cm), two applied voltages for each NG (applied voltages were selected based on the stability of the spray, i.e. lower and upper voltages of the stable cone-jet mode for each NCD: at 5 cm, 12 and 13 kV for 25G, 9 and 11 kV for 27G, and 8 and 12 kV for 30G; at 10 cm, 15.5 and 16.5 kV for 25G, 12 and 15 kV for 27G, and 12 and 16 kV for 30G), and four flow rates (FR) (1, 2, 5, and 7 mL/h). A *n* = 5 was considered for each group and for each electrospraying parameter test.

#### Viability and morphology

Collected samples were then incubated for 24 h, after which chondrocyte viability was assessed using resazurin reduction assay. Briefly, a resazurin solution (0.1 mg/mL; ACROS Organics) in PBS was added to culture medium at a final concentration of 10 % (v/v), and chondrocytes were incubated in this solution at 37°C for 4 h in the dark, after which 100 µL per well was transferred to a 96-well plate and absorbance at 570 and 600 nm was measured. The final absorbance values for each sample were calculated as the ratio Abs570/Abs600 nm minus the Abs570/Abs600 nm ratio of a negative control (culture medium). The absorbance values of CC were then taken as 100% and cell viability calculated as a percentage of these control values. A live/dead assay was also used to assess cell viability. At each time point, culture medium containing 0.25 µM Calcein-AM (Invitrogen) and 30 µM propidium iodide (Sigma-Aldrich) was added to the samples. After 25 min of incubation in the dark at 37°C, in 5 % CO_2_, samples were subsequently washed with Hanks’ Balanced Salt Solution (Gibco), and imaged using a fluorescence microscope (Axioimager M2, Zeiss) with a magnification of 10x/0.25. A dead control, in which ethanol was introduced to the chondrocytes prior to staining was also investigated. The generated pictures (*n =* 3 per condition) were processed with Fiji software to quantify the regions positively stained for each marker in relation to the overall image area. These data were used to determine the viability of the chondrocytes following electrospray treatment. The morphology of chondrocyte was visualized using an inverted optic microscope (Euromex, CMEX-PRO 10MP; The Netherlands).

#### Proliferative behavior

The proliferative ability of the electrosprayed C28/I2 chondrocytes subjected to different NG and NCD parameters was assessed. Briefly, 2 × 10^4^ electrosprayed C28/I2 chondrocytes were seeded in 48-well plates and cultured over a 14-day culture period, where medium changes were also performed two times a week. At days 1, 7, and 14, chondrocyte viability and morphology were once more assessed as previously described.

#### Gene expression

Potential alterations in post-electrosprayed C28/I2 gene expression, using different NG (25G, 27G, and 30G) and NCD (5 and 10 cm), was evaluated using fluorescence-base quantitative (real-time) PCR. Total RNA was purified from approximately 5 × 10^5^ cells using Quick-RNA™ MicroPrep Kit (50 Preps) w/Zymo-Spin™ IC Columns (Zymo) or RNeasy^®^ Micro (Quiagen) according to the manufacturer’s instructions. A *n* = 3 was considered for each condition. RNA quality and concentration was evaluated using a NanoDrop^®^ spectrophotometer. First strand cDNA was generated from 250 μg of total RNA using the NZY First-Strand cDNA Synthesis Kit (Nzytech) following the manufacturer’s instructions.

*COL2A1* (Collagen Type II Alpha 1 Chain), *COL1A1* (Collagen Type I Alpha 1 Chain), and *ACAN* (Aggrecan) forward and reverse primers were designed using Primer-BLAST (NCBI) and verified for secondary structure using Beacon Designer™ Free Edition, *HPRT1* forward and reverse primers sequence was obtained from a previous publication.^43^ Two microliter of each cDNA was added to a mixture containing 1X NZYSpeedy qPCR Green Master Mix (Nzytech) and 300 nM of primers (primer sequences and amplification efficiency, Supplementary Table S1) in 96-well plates. Samples and no template control were run in duplicate. A melting curve was generated for all genes and random samples were analyzed by gel electrophoresis (2%) to confirm the specificity of the assays. *HPRT1* was validated and used as reference gene. Relative gene expression was calculated using the Livak method.^44^

### Combined C28/I2 chondrocyte electrospraying and polymer electrospinning

#### Solutions

The polymeric solution preparation followed a protocol previously reported.^45^ Briefly, PCL (Sigma-Aldrich; 80 kDa) and GEL (from porcine skin; Sigma-Aldrich) were dissolved separately in 2,2,2-trifluoroethanol (TFE; TCI) at a concentration of 10% and stirred vigorously at room temperature for 12–18 h. Before electrospinning, the two solutions were mixed in 50:50 volume ratios (PCL + GEL) with 0.2% acetic acid (Sigma-Aldrich), filtered-sterilized with a 0.45 µm pore size filter of polytetrafluoroethylene inside the laminar flow hood and poured into a plastic syringe. 1 × 10^6^ C28/I2 chondrocytes were suspended in 300 µL of culture medium and poured into another plastic syringe.

#### Electrospinning and electrospraying process

Both the polymeric solution, the chondrocyte suspension and respective tubing’s were placed in the NANON 01 electrospinning machine, as illustrated and depicted in Supplementary Figure S1a and b, respectively. Afterwards, first a layer of PCL + GEL was electrospun for nearly 10 min at 1.5 mL/h at 27 kV through a 21G needle (0.51 mm diameter and 1.5 mm length). The needle was placed 10 cm above a sterile petri dish with a sheet of aluminum foil connected to the ground through a copper wire. After spinning a layer of PCL + GEL, the C28/I2 chondrocyte suspension was electrosprayed onto the polymer layer for 4 min at 2 mL/h at 9 kV through a 27G needle at a needle to collector distance of 5 cm. Subsequently, PCL + GEL was electrospun again for another 10 min at 1.5 mL/h followed by C28/I2 chondrocyte electrospraying for 4 min. A final PCL + GEL layer was electrospun on top of the construct to seal the chondrocytes into the construct. The final construct, shown in Supplementary Figure S1c, consisting of five alternating layers of PCL + GEL (three electrospun layers) and chondrocytes (two cell layers) with an approximate thickness of 100 µm, was then detached from the aluminum foil, cut into 15 mm squares and placed in 24-well plates. Samples were then incubated in DMEM/F-12 supplemented with 10% FBS, 1% P/S, and 0.25 µg/mL Amphotericin B for 7 days in 5% CO_2_ at 37°C. The medium was refreshed two times a week. A *n = 3* was considered for these experiments.

#### Constructs characterization

Viability measurements were performed via a resazurin assay, as previously described. The viability of post-electrosprayed chondrocytes was calculated as a percentage of the CC values—chondrocytes that were not subjected to the electrospraying process. A cytochemical staining of the nuclei was used to visualize cells in the constructs from a top view perspective after 7 days of culture. After fixation with 4% paraformaldehyde (ACROS Organics) in PBS and permeabilization with 0.1% v/v Triton X-100 (Fisher Scientific), cells were stained for nuclei (4′,6-diamidino-2-phenylindole, DAPI; Sigma-Aldrich) and then visualized using a fluorescence microscope (Axioimager M2, Zeiss) with magnification of 20x/0.50. In order to visualize a chondrocyte’s layer, the final PCL + GEL layer was detached from the constructs, and the remaining parts were dehydrated with increasing concentrations of ethanol aqueous solutions (50, 70, 90, 95, and 100% v/v), treated with hexamethyldisilane (HMDS; TCI), kept overnight in a fume hood for air drying, mounted in an aluminum stub, and observed by Scanning Electron Microscopy (SEM) using a Hitachi TM4000 plus (Japan) at an accelerating voltage of 10 kV.

### Electrospraying of primary chondrocytes

Primary cell suspensions were split into two groups, each with 1 × 10^6^ chondrocytes suspended in 300 µL of culture medium with 0.25 µg/mL Amphotericin B: culture control (CC), which were maintained in the laminar flow hood at room temperature during the electrospraying process; and electrosprayed samples (*E*), where the cell suspensions were pumped through the electrospraying apparatus and exposed to voltage. A *n =* 3 was considered for each group. These chondrocyte suspensions were also electrosprayed into a copper-wired well of a 24-well plate with culture medium, using a 27G needle combined with a needle to collector distance of 5 cm, a flow rate of 2 mL/h, and an applied voltage of 9 kV. Collected samples and the respective controls were also then incubated at 37°C in a humidified atmosphere of 5% CO_2_ in air. Afterwards, chondrocyte viability and morphology were assessed as previously described.

### Statistical analysis

All the quantitative data are expressed as mean ± standard deviation. Statistical significance was determined, using OriginLab, by performing as suited One-way analysis of variance (ANOVA), One-way ANOVA with repeated measures, and Two-way ANOVA, all followed by post hoc Tukey’s test. Statistical significance was also determined Kruskal-Wallis One Way, when suited. Significance was accepted at *p*-values inferior to 0.001, 0.01, and 0.05.

## Results

### Establishment of electrospray stability

**Figure 2.**
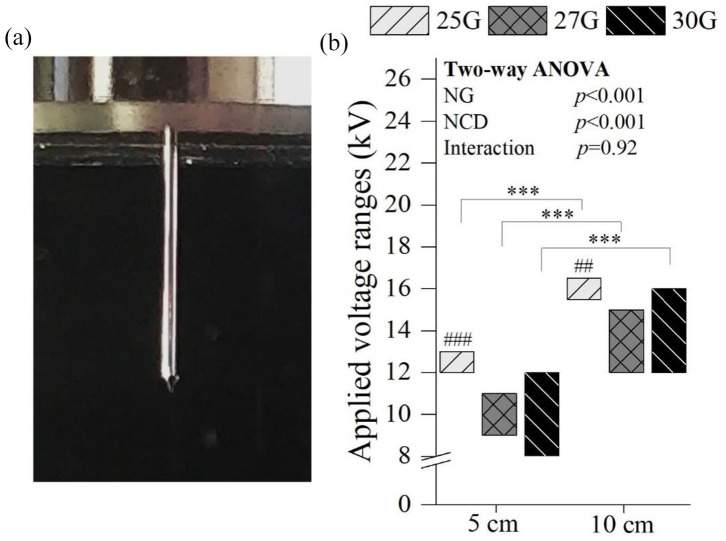
Stable cone-jet mode clearly depicting the Taylor cone (a) and the applied voltage ranges where a stable cone-jet mode was observed (b). One parameters was constant: 2 mL/h. Statistical analysis by Two-way ANOVA followed by post hoc Tukey’s test; ***p < 0.001, ###*p* < 0.001, ##*p* < 0.01, where * denotes statistical significant differences between different NCD for each NG, while # denotes statistical significant differences between different NG for each NCD.

The stability of electrospray was evaluated by macroscopic visualization by the presence of the Taylor cone in the spray formed by applying a range of voltages to the solution droplet, as depicted in Figure 2. Stable cone-jet modes at 5 cm NCD were observed for 25G between 12 and 13 kV, for 27G between 9 and 11 kV, and for 30G between 8 and 12 kV. At 10 cm, higher voltages were necessary to obtain a stable cone-jet mode (*p* < 0.001), for all NG; indeed, for 25G this mode was observed between 15.5 and 16.5 kV, for 27G between 12 and 15 kV, and for 30G between 12 and 16 kV. Below the lower voltage of each range, no spray was produced, only droplets fell from the needle. Above the upper voltage value of each range reported, the spray was irregular and unstable, causing discontinuous jetting. A statistically significant difference was found between the voltage range of the 25G needle with the other NG for both NCD (*p* < 0.001 at 5 cm and *p* < 0.01 at 10 cm). Also, wider stability voltage ranges were obtained with decreasing NG.

### Impact of electrospraying parameters on C28/I2 chondrocyte viability

#### Electrospraying process and needle gauge

**Figure 3.**
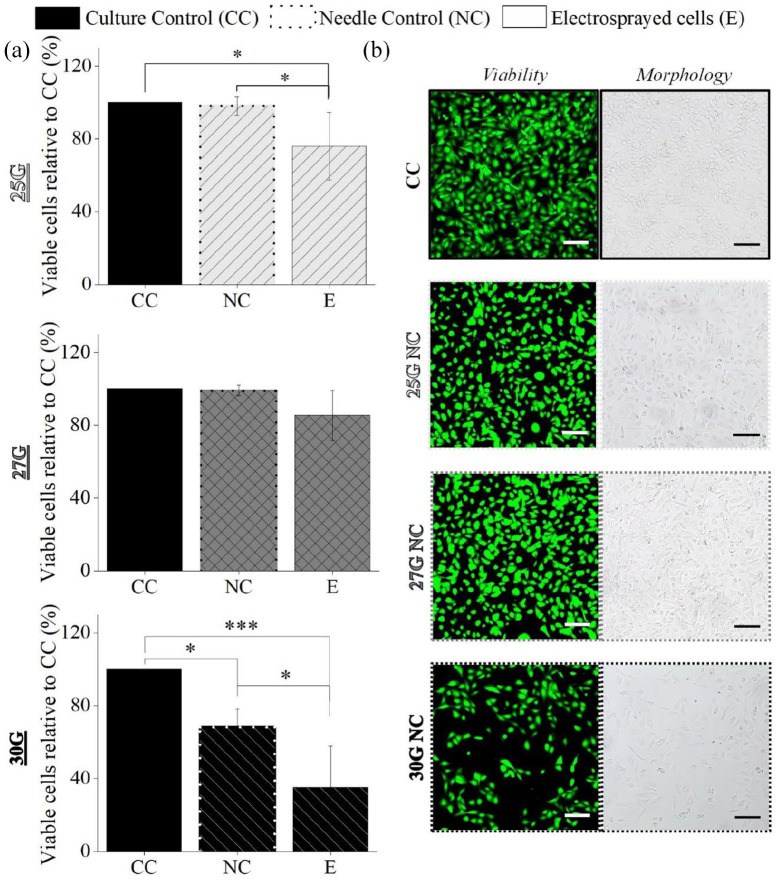
Influence of the electrospray process on the percentage of viable post-electrosprayed chondrocytes after 24 h using 25, 27, and 30G needles with and without voltage (a) and the live/dead staining images (green corresponds to live cells and red to dead cells) and optical micrographs of the culture and needle controls (b). Two parameters were constant: 2 mL/h and 5 cm NCD. Applied voltages: 12 kV for 25G, 9 kV for 27G, and 8 kV for 30G. Scale bars: 100 µm. Statistical analysis by One-way ANOVA followed by post hoc Tukey’s test: ***p < 0.001, *p < 0.05.

A statistically significant difference on the percentage of viable chondrocytes between CC and E groups was found for 25G (76% ± 18%; *p* < 0.05) and for 30G (35% ± 22 %; *p* < 0.001) (Figure 3(a)). Moreover, using a 30G needle, a reduction of the E cells’ number was also observed in the NC in comparison with CC (68% ± 10 %; *p* < 0.05). This was confirmed by live/dead staining and quantification, which revealed a two-fold reduction in the live covered area between the CC (52.21% ± 8.10 %) and 30G NC groups (23.18 ± 5.87 %, *p* < 0.05) (Supplemental Figure S2). Nonetheless, no difference in viability was seen between the CC and NC groups when 25G (98% ± 5 %) and 27G (99% ± 2 %) needles were used (Figure 3(a)). A similar pattern was seen in the quantification of live/dead cells (55.49% ± 9.58 % for 25G and 52.81% ± 13.09 %) (Supplemental Figure S2). There were no noticeable variations in chondrocyte morphology between the CC and NC groups in terms of viability. Additionally, the apparent number of visible chondrocytes on micrographs and live/dead staining images matched the viability data (Figure 3(b)).

#### Applied voltage

**Figure 4.**
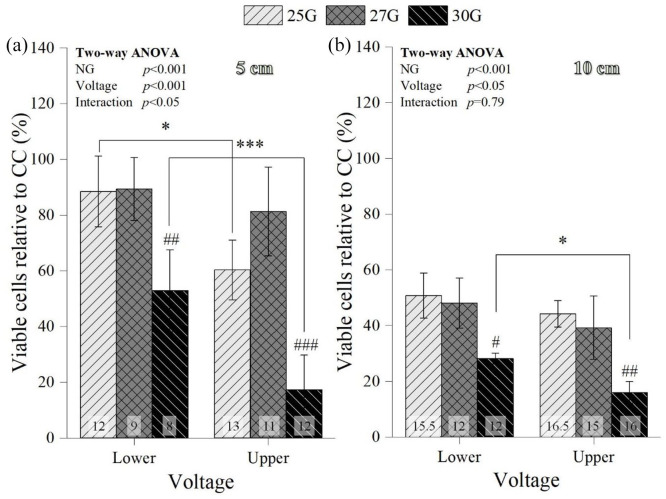
Influence of the voltage on the percentage of viable post-electrosprayed chondrocytes after 24 h using a needle to collector distance of 5 cm (12 and 13 kV for 25G, 9 and 11 kV for 27G, and 8 and 12 kV for 30G; (a)) and 10 cm (15.5 and 16.5 kV for 25G, 12 and 15 kV for 27G, and 12 and 16 kV for 30G; (b)). One parameter was constant: 2 mL/h. Statistical analysis by Two-way ANOVA followed by post hoc Tukey’s test; ^###^*p* < 0.001, ^##^*p* < 0.01, ^#^*p* < 0.05, ***p < 0.001, *p < 0.05, where * denotes statistical significant differences between different applied voltages for each needle diameter, while # denotes statistical significant differences between different needle diameters for each voltage.

Increasing the applied voltage at 5 cm—within the stable cone-jet mode—generated a considerable reduction of the viable E chondrocytes’ percentage, when 25G (from 88% ± 12% to 60% ± 11%; *p* < 0.001)—from 12 to 13 kV—, and 30G (from 53% ± 15% to 17% ± 12%, *p* < 0.001)—from 8 to 12 kV—, needles were used (Figure 4(a)). On the contrary, no statistically significant differences were observed on chondrocyte viability using a 27G NG (from 89% ± 11% to 81% ± 16 %) increasing from 9 to 11 kV. These results are corroborated by the apparent number of chondrocytes visible on the micrographs, which was considerably lower using a 30G needle (Supplemental Figure S3). At 10 cm, a similar behavior was observed for the 30G needle (28% ± 2% to 16% ± 4%, *p* < 0.05; Figure 4(b))—increasing from 12 to 16 kV—, while for the 25G (50% ± 8% to 44% ± 5%) and 27G (48% ± 9% to 39% ± 11%) needles, no significant differences were found increasing the applied voltage from 15.5 to 16.5 kV for 25G and from 12 to 15 kV for 27G (Figure 4(b)).

#### Needle to collector distance

**Figure 5.**
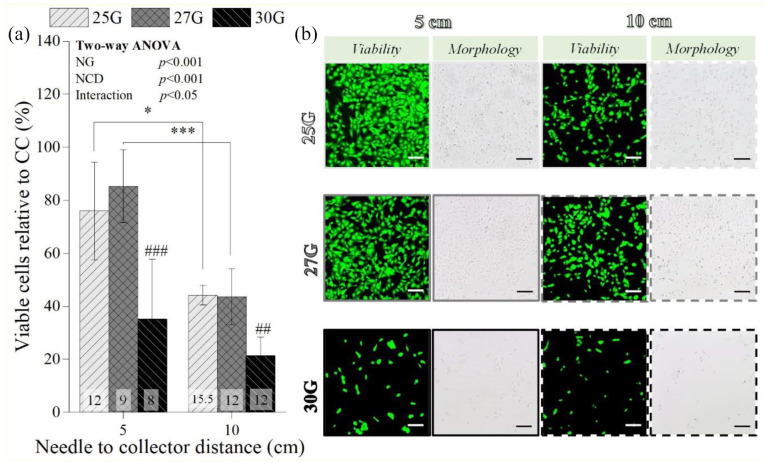
Influence of the needle to collector distance on the percentage of viable post-electrosprayed chondrocytes after 24 h (a) and the respective live/dead staining images (green corresponds to live cells and red to dead cells) and micrographs (b). One parameter was constant: 2 mL/h. Applied voltages: 12 kV for 25G, 9 kV for 27G, and 8 kV for 30G at 5 cm NCD; and 15.5 kV for 25G, 12 kV for 27G, and 12 kV for 30G for 10 cm NCD. Scale bars: 100 µm. Statistical analysis by One-way ANOVA followed by post hoc Tukey’s test: ^###^*p* < 0.001, ^##^*p* < 0.01, ***p < 0.001, *p < 0.05, where * denotes statistical significant differences between different NCD for each needle diameter, while # denotes statistical significant differences between different needle diameters for each NCD.

A higher NCD (10 cm) substantially lowered the number of viable chondrocytes when 25G (from 76% ± 18% to 44% ± 3%; *p* < 0.05) and 27G (from 85% ± 14% to 44% ± 11 %; *p* < 0.001) were employed, while for 30G NG group no statistically significant differences were found between the tested NCD (Figure 5(a)). This was validated by the live/dead area quantification (from 54.72% ± 5.59% to 21.02% ± 3.74 % for 25G (*p* < 0.001); from 51.80% ± 3.91% to 21.25% ± 4.01% for 27G (*p* < 0.001)) (Supplemental Figure S4). Furthermore, when the 30G was used, post-electrosprayed samples had a significantly lower percentage of viable chondrocytes (35 ± 22% at 5 cm, *p* < 0.001; and 21 ± 7% at 10 cm, *p* < 0.01, Figure 5(a)), as confirmed by the live/dead quantification (6.61 ± 1.75 at 5 cm, *p* < 0.001; and 3.40 ± 1.26% at 10 cm, *p* < 0.001; Supplemental Figure S4). These results are in agreement with the lower number of chondrocytes visible in the live/dead staining images and micrographs of the samples electrosprayed at 10 cm and with 30G (Figure 5(b)).

#### Flow rate

**Figure 6.**
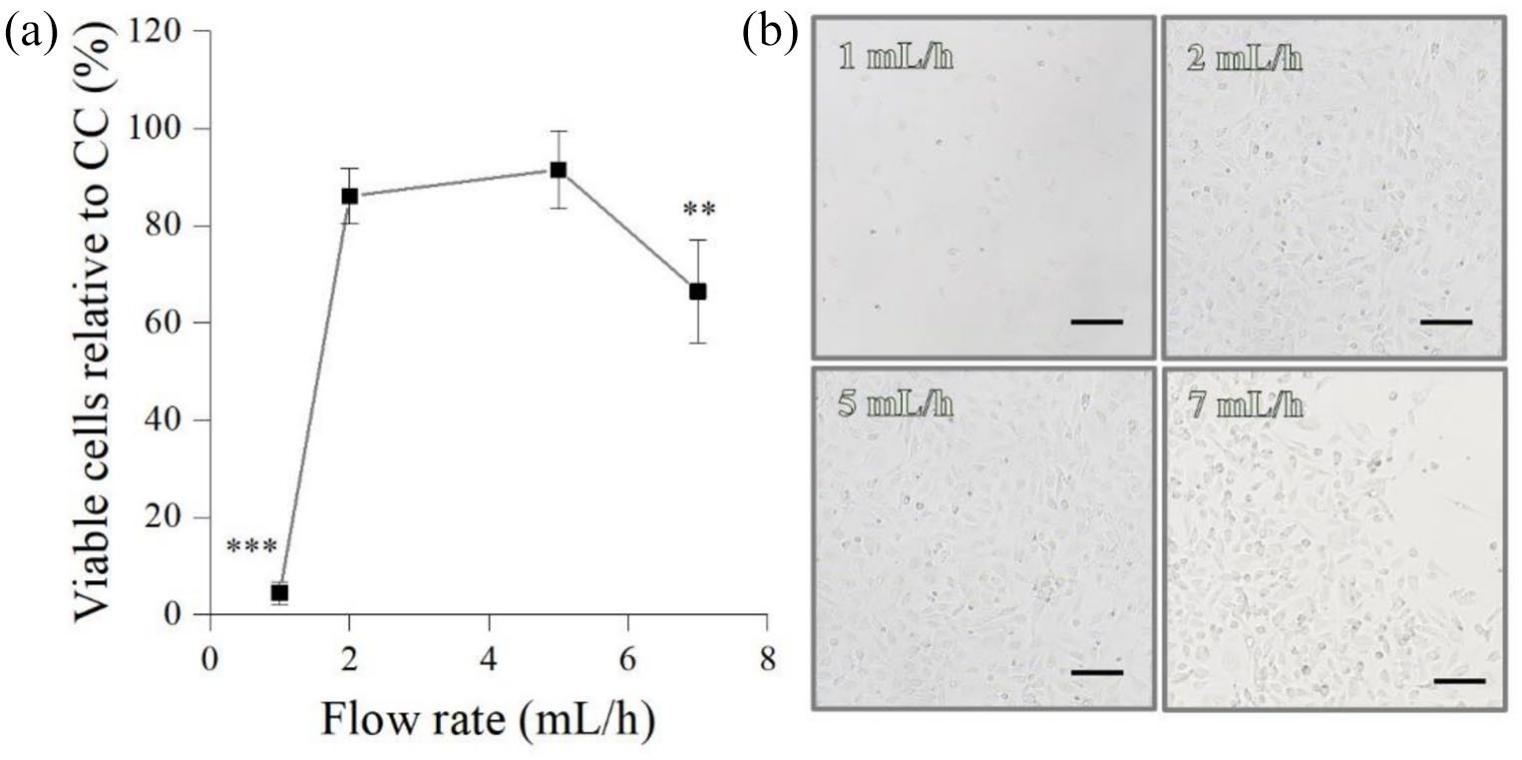
Influence of flow rate on the percentage of viable post-electrosprayed chondrocytes after 24 h (a) and the respective micrographs (b). Three parameters were constant: 27G needle, 9 kV and 5 cm NCD. Scale bars: 100 µm. Statistical analysis by One-way ANOVA followed by post hoc Tukey’s test; ***p < 0.001, **p < 0.01.

FR’s impact on E C28/I2 chondrocytes was also assessed for a constant NG (27G), applied voltage (9 kV), and NCD (5 cm) (Figure 6(a)). About 2 (86% ± 6 %) and 5 (91% ± 8%) mL/h allowed substantially higher number of viable post-electrosprayed chondrocytes, whereas 1 mL/h resulted in extensive chondrocyte death (4% ± 2%, *p* < 0.001; Figure 6(a)), which is also consistent with the fewer chondrocytes exhibited in the micrographs (Figure 6(b)). Likewise, 7 mL/h also generated a substantial reduction on the percentage of viable chondrocytes (66% ± 10 %; *p* < 0.01).

### Influence of the electrospraying parameters on C28/I2 chondrocyte gene expression

**Figure 7.**
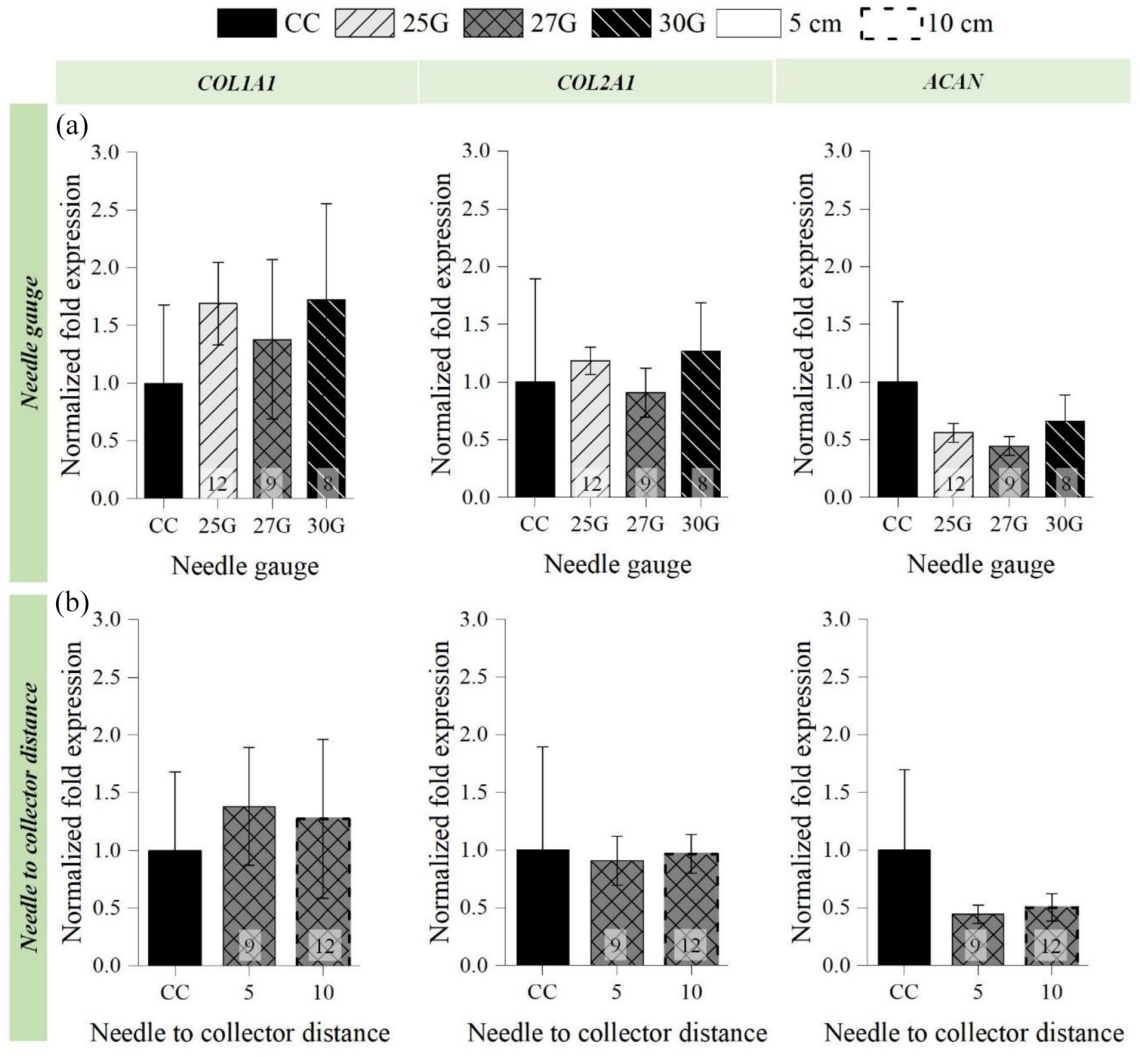
Influence of the needle gauge (a) and needle to collector distance (b) on the *Col1A1*, *Col2A1*, and *ACAN* expression by post-electrosprayed chondrocytes after 24 h. Constant parameters: (a) 2 mL/h and 5 cm NCD; (b) 27G needle and 2 mL/h. Applied voltages: (a) 12 kV for 25G, 9 kV for 27G, and 8 kV for 30G; (b) 9 kV for 5 cm and 12 kV for 10 cm. Statistical analysis by Kruskal-Wallis One Way for *Col1A1* expression (a) and by One-way ANOVA for *Col1A1* (b), *Col2A1*, and ACAN expression.

No statistically significant differences were detected after 24 h on gene expression of chondrocytes electrosprayed at 5 cm NCD using different NG and the respective culture control chondrocytes gene expression (COL1A1 (*p =* 0.58), COL2A1 (*p =* 0.82), and ACAN (*p =* 0.35) (Figure 7(a)). Likewise, COL1A1 (*p =* 0.76), COL2A1 (*p =* 0.82), and ACAN (*p =* 0.27) expression on chondrocytes electrosprayed using different NCD did not displayed significant differences with the culture control (Figure 7(b)).

### Influence of the electrospraying parameters on C28/I2 chondrocyte long-term proliferative behavior

**Figure 8.**
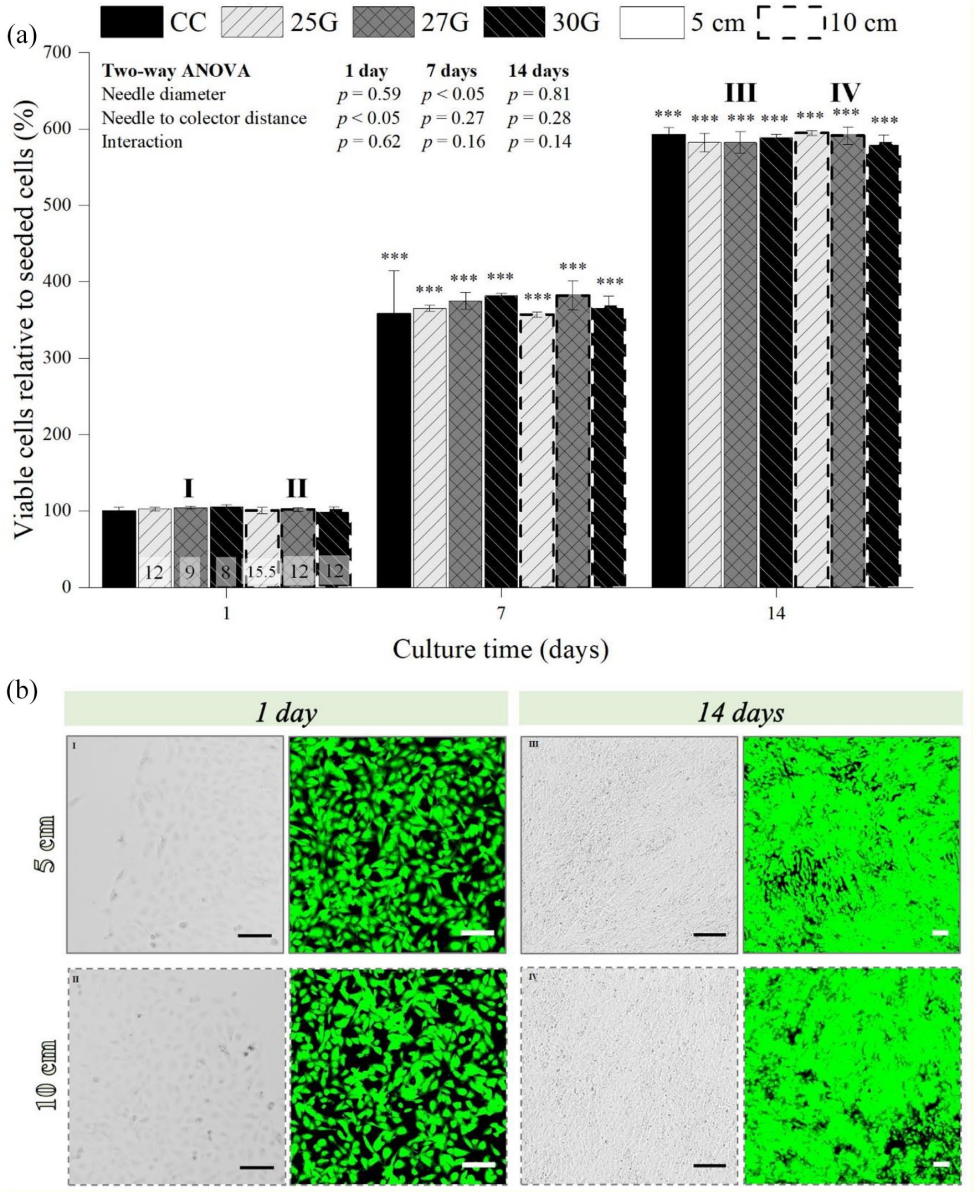
Influence of the electrospraying parameters (needle diameter—25, 27, and 30G—and needle to collector distance—5 and 10 cm) on the long-term viability of post-electrosprayed chondrocytes (2 × 10^4^ electrosprayed C28/I2 chondrocytes were exposed to each condition combination of needle diameter and needle to collector distance), cultured for 14 days (a) and the micrographs and live/dead staining images (green corresponds to live cells and red to dead cells) relative to the 27G needle. One parameter was constant: 2 mL/h. Applied voltages: 12 kV for 25G, 9 kV for 27G, and 8 kV for 30G at 5 cm NCD; and 15.5 kV for 25G, 12 kV for 27G, and 12 kV for 30G for 10 cm NCD. Scale bars: 100 µm. Statistical analysis by Two-way ANOVA followed by post hoc Tukey’s test; by One-way ANOVA with repeated measures followed by post hoc Tukey’s test; ***p < 0.001, where * denotes statistical significant differences different needle diameters and needle to collector distance condition combination over culture time.

The proliferative behavior of the electrosprayed C28/I2 chondrocytes (2 × 10^4^ electrosprayed C28/I2 chondrocytes subjected to each NG and NCD condition combination) was then assessed over a culture period of 14 days, where a significant increase on the percentage of viable post-electrosprayed chondrocytes was observed over time for all the NG and NCD combinations (*p* < 0.001; Figure 8(a)). This behavior was also detected not only on the chondrocyte micrographs, where substantially more cells were found with increasing culture time, but also in live/dead staining images and the respective quantification (Figure 8(b), Supplemental Figures S5 and S6). After 1 day of culture, significant differences were found between the NCD employed (*p* < 0.05). At day 7, statistically significant differences were found on viable chondrocyte percentage between 25 and 27 NG for both NCD tested (*p* < 0.05). Yet, by the end of the culture period no significant differences were observed between the number of the viable E chondrocytes subjected to all the parameters permutation (Figure 8(a)). Furthermore, the viability and morphological studies were indistinguishable between the CC and E samples, regardless of the NG and NCD combination (Figure 8(b), Supplemental Figures S5 and S6).

### Combined C28/I2 chondrocyte electrospraying and PCL + GEL electrospinning approach

**Figure 9.**
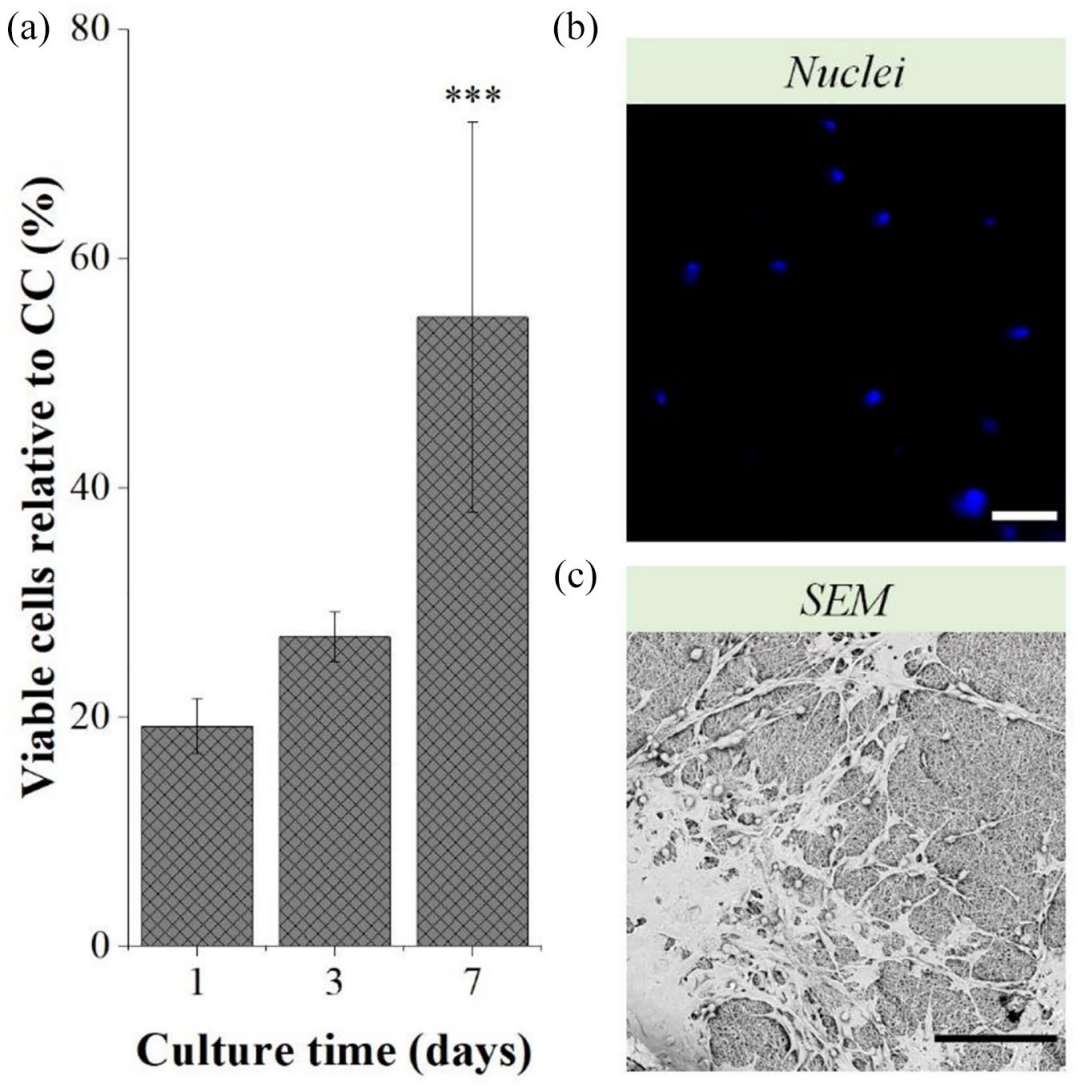
Percentage of viable C28/I2 chondrocytes relative to CC—non-electrosprayed chondrocytes—of the constructs after 1, 3, and 7 days of culture (a) and the respective immunocytochemistry images of the nuclei of chondrocytes embedded in the PCL+GEL fibers (b), and SEM images of the chondrocytes’ layer after 7 days of culture (c). Scale bars: 100 μm. Statistical analysis by One-way ANOVA with repeated measures followed by post hoc Tukey’s test; ***p < 0.05.

The percentage of viable C28/I2 chondrocytes embedded in the PCL + GEL fibers after the multi-layered approach was considerably low after 1 day of culture (19.17% ± 2.43 %; Figure 9(a)). Nevertheless, over a 7-day culture period, there was a significant increase of this percentage, from 26.99% ± 2.14% at day 3 to 54.86 ± 17.03% at day 7 (*p* < 0.001). Indeed, after 7 days, it was possible to find chondrocytes embedded in PCL + GEL fibers (Figure 9(b)), even though fibers’ autofluorescence did not allow a clear visualization of the nuclei. So, a layer of fibers was detached from the construct to allow the visualization of a chondrocyte layer and the resulting SEM image is displayed in Figure 9(b). C28/I2 chondrocytes appeared well attached to the fibers, displaying a similar morphology to the cells only subjected to the electrospraying process. Furthermore, their distribution along the construct was fairly homogeneous.

### Primary chondrocytes electrospraying

**Figure 10.**
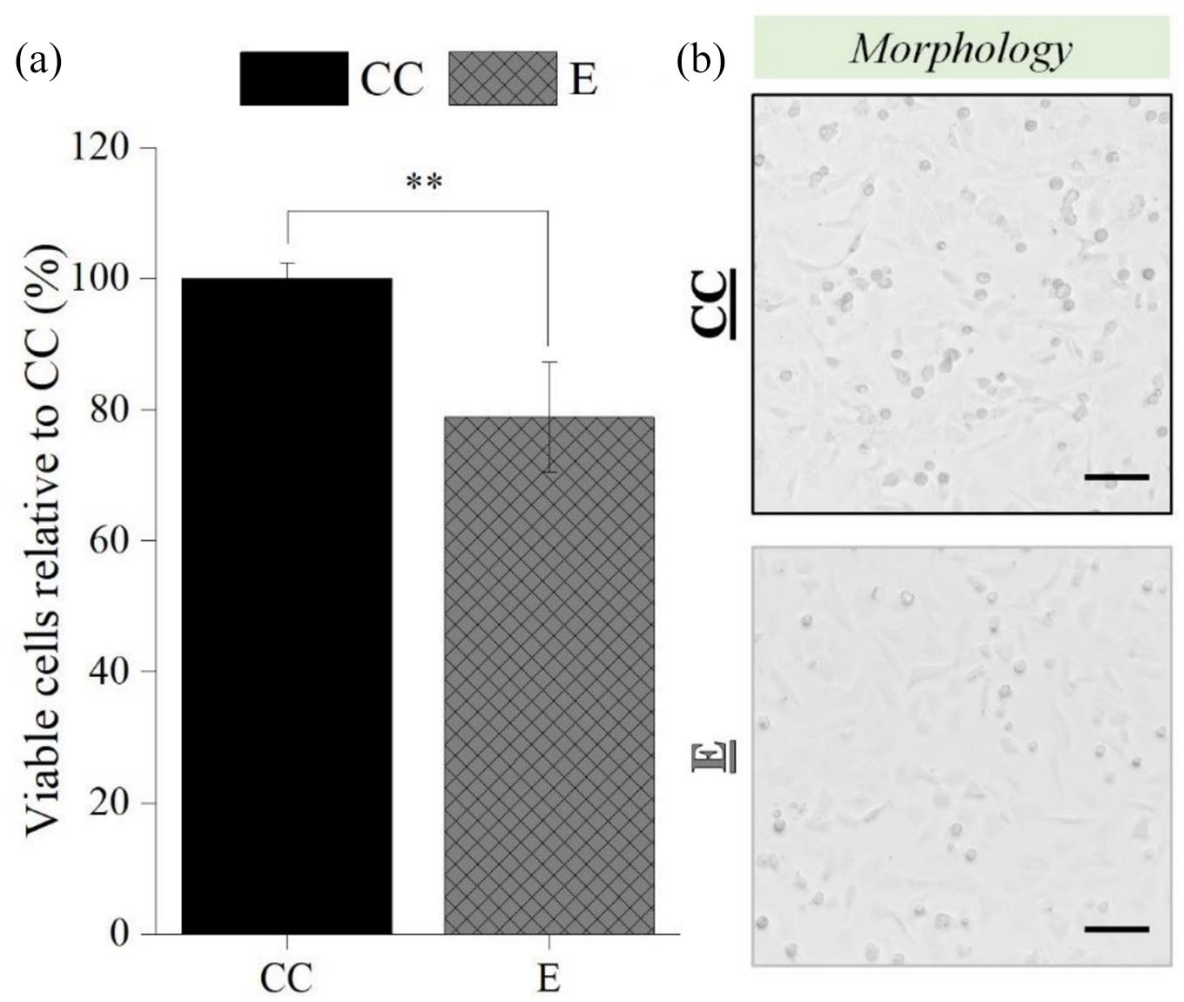
Influence of the electrospraying process on the percentage of viable post-electrosprayed primary chondrocytes after 24 h (a), and the respective micrographs (b). Electrospraying parameters: 27G needle, 2 mL/H, 5 cm NCD, and 9 kV. Scale bars: 100 µm. Statistical analysis by One-way ANOVA followed by post hoc Tukey’s test, **p < 0.01.

Once electrospraying process was optimized, the optimal parameters were subsequently employed to perform electrospraying of primary cells: 27G needle, 9 kV, 2 mL/h, and 5 cm NCD, and the results are displayed in Figure 10. A statistically significant difference on the percentage of viable chondrocytes between CC (100% ± 2.36 %) and E (78.85% ± 8.37%) groups was found (*p* < 0.01; Figure 10(a)), which was corroborated by the apparent number of chondrocytes visible on the micrographs (Figure 10(b)). Nevertheless, chondrocyte maintained a rounded to polygonal morphology upon exposure to the electric field (Figure 10(b)).

## Discussion

In an attempt to fabricate homogeneous and functional TE constructs, several reports have explored bio-electrospraying as an alternative for conventional cell seeding techniques in electrospun scaffolds.^17,46,47^ Yet, for chondrocyte electrospraying to be effectively employed for cartilage TE, it is of the utmost importance to assess if chondrocytes are in any way adversely affected. So, the present work seeks to understand the influence of the electrospraying technology on chondrocyte viability and function, as well as the establishment of optimal operational parameters for maximum chondrocyte viability.

First, and since this technology is to be used for the precise and uniform cell placement in 3D architectures for TE constructs,^17,18^ jet stability should be achieved. Unlike previous reports,^19,26,30^ it was possible to electrospray chondrocyte suspensions in a stable cone-jet mode, regardless of the NG and NCD. While it has been suggested that the high conductivity and low viscosity of the cell suspension may contribute to spray instability,^26^ it is also considered that the nozzle geometry—in this case NG, electrode configuration and flow rate (FR)—all play a significant role in achieving a stable cone-jet mode.^22^ Indeed, the combination of a smaller NG, a higher NCD and a higher FR may have been a deciding factor in this case.

Upon the establishment of a stable spray, chondrocyte viability was evaluated for the variation of each electrospraying operational parameter. From the three NG tested, only 30G had a detrimental effect on chondrocytes—NC. It is possible that chondrocyte shearing whilst passing through the needle, particularly using 2 mL/h, was the reason for this effect, which is consistent with previous reports.^32,48^ This chondrocyte mortality was exacerbated upon exposure to the electric field. A similar reduction on post-electrosprayed chondrocyte viability was observed for the 25G NG, while with 27G NG no significant harmful influence was observed. It is possible that the higher voltages required for spray stability on 25G needle had a somewhat adverse impact on the chondrocyte metabolism. As a matter of fact, increasing the system applied voltage systematically reduced chondrocyte viability. It has been suggested that high voltages, that generate strong electric fields, can induce pore formation and cell membrane damage, followed by an increased membrane permeabilization and, consequent cellular osmotic imbalance, ultimately resulting in cell death.^38,39,49^ Moreover, beside electrical damages, strong electric fields can also incite thermal damage on the cells.^39,49^ Interestingly, this damage was not detected on the chondrocytes electrosprayed through a 27G needle, except when NCD was increased. In fact, a significant viability reduction was detected when chondrocytes were electrosprayed at 10 cm. Given that higher voltages are required to maintain the electric field intensity at increasing NCD, the chain of events described before may have contributed to cell death. Other authors have associated this decrease to other occurrences. Paletta et al.^38,48,50^ perceived an increased evaporation rate of the cell-laden droplets at higher NCD, ultimately resulting in an increased salt concentration, and therefore, reduced cell survival.^14^ Several authors have attributed greater cell loss to higher NCDs. This cell loss could explain why no dead cells were visible on the live/dead staining images, regardless of the conditions used.

Regarding FR, it was possible to narrow the optimal values for maximum chondrocyte viability from 2 to 5 mL/h, particularly using a 27G NG and 5 cm NCD. Above 5 mL/h, shear stresses played a significant role on chondrocyte mortality.^32,48^ Below 2 mL/h, it is believed that chondrocyte death was mainly due to the electrospraying duration. Indeed, electrospraying time using 1 mL/h was 18 min, while pumping at 2 and 5 mL/h only 9 and 4 min were necessary, respectively. Besides the longer high voltages’ submission time, the prolonged exposure to lower temperatures (~25°C–27°C) and CO_2_ concentration (~0.04%) may have contributed to chondrocyte death.^14,38^ Actually, Braghirolli et al.^38^ performed an evaluation on electrosprayed cells with different electrospraying times and found that, while no differences were detected on cell viability, there were breaks in the DNA on the samples subjected to longer electrospraying periods (30 and 60 min), indicating that prolonged electrospraying periods of time might provoke cellular genotoxicity. Several reports have suggested the inclusion of a polymeric hydrogel onto the cell suspension in order to increase its viscosity, and reduce the impact of high voltages, dehydration, and environmental conditions.^15,17,50,51^

Regardless of the electrospraying parameter permutation, electrosprayed chondrocyte were still able to attach to the tissue culture polystyrene and present their typical rounded to polygonal morphology.^52^ Moreover, the percentage of viable chondrocytes submitted to certain electrospraying (27G NG, 9 kV, and 5 cm NCD) parameters remained high—above 70%. It should also be emphasized that while other studies have reported higher post-electrosprayed cell viabilities (above 80%–90%),^32,38,39,47,53^ it is important to mention that most of these employed substantially bigger NG and smaller NCD, which according to the herein reported data should render high viabilities. Additionally, different electrospraying conditions, viability assay sensitivity and cell susceptibility to damage may also be responsible for the observed difference.^14,39^

Interestingly, despite the fact that short-time viability assays disclosed detrimental effects of several electrospraying parameters, no significant differences in the expression levels of chondrogenic genes between post-electrosprayed chondrocytes and their respective CC were observed, regardless of the electrospraying parameters used. Collagen type II and aggrecan were selected because they are the major components of hyaline cartilage which includes articular cartilage. Both molecules are produced by chondrocytes and released to the extracellular matrix.^54,55^ On the other hand, collagen type I is not normally present in hyaline cartilage, but it is produced when chondrocytes dedifferentiate in vitro or in vivo in the context of osteoarthritis and other arthritic diseases. Thus, the preservation of collagen type II and aggrecan expression, observed here, is an indicator that the cells are functional and maintain the phenotypic characteristics of mature chondrocytes.^56,57^ Gene expression was assessed after 24 h of electrospraying. Prior work has found that expression of type II collagen and aggrecan could be detected only 2 h after chondrocyte deposition.^58^ Furthermore, several reports have also selected this time point.^59,60^

The long-term proliferation studies also revealed that no obvious differences between each parameter permutation and the respective CC were found in terms of gross morphology and rate of growth to confluence. This might imply that the decreased vitality of chondrocytes was mostly due to chondrocyte loss within the electrospraying chamber. These results further suggest bio-electrospraying under the optimal operational conditions allows the successful delivery of chondrocytes. Following that, combining this technology with polymer electrospinning, in this case via an alternated chondrocyte electrospraying and polymer electrospinning approach—combining lower and higher NCD, respectively—may be a promising strategy for maximizing chondrocyte survival while maximizing polymer solvent evaporation. This “cell layering” approach has been already reported with successful cell incorporation, although bio-electrospraying was not always employed in this instance.^16,17,61^

To investigate this possibility, we conducted a straightforward experiment that validated this concept. In this case, an alternated electrospinning and electrospraying approach was required not only due to the different NCD, but also due to the different voltage requirements for each process—10 cm and 27 kV for PCL + GEL electrospinning and 5 cm and 9 kV for C28/I2 chondrocyte electrospraying, which could not be conducted simultaneously due to the equipment limitations. The results obtained in this study demonstrated that chondrocyte integration was achievable when electrospraying and electrospinning were combined, and that their distribution within the PCL + GEL fibers was fairly homogeneous. However, these findings were not yet optimal due to the low initial chondrocyte viability, which can be attributed mainly to experiment’s duration and chondrocyte dehydration. Still, solvent toxicity, in this instance, might have been the determinant factor. Indeed, the choice of the polymer solvent is critical, as residues left in the polymer fibers could be cytotoxic.^16,62^ Canbolat et al. found that using hexafluoro-2-propanol as PCL solvent allowed 80% cell attachment, while only 50% cell attachment was found using dimethylformamide (DMF) and chloroform.^16^ TFE, like DMF and chloroform, is highly toxic. Moreover, several studies have reported the TFE toxicity in vivo in rats.^63,64^ So, residual TFE left in the electrospun fibers might be detrimental for the cells, especially if no washing step can be performed. In fact, Nam et al.^62^ has reported chondrocyte toxicity with fluorinated alcohols concentration of 500 ppm or higher and, upon chondrocyte seeding, cellular growth and proliferation might be delayed or limited until the solvent is completely removed, explaining why chondrocyte viability increased considerable after 7 days of culture. These preliminary results highlight the need for additional optimization studies. There are reports of increased cell survival when employing a partly liquid collector and non-toxic solvent systems,^61^ suggesting that these hypothesis should be considered in future investigations. Nonetheless, the percentage of viable C28/I2 chondrocytes increased during the course of the culture, indicating that the chondrocytes embedded within the PCL + GEL fibers retained their proliferative capacity. Given that electrospinning can replicate not only the hierarchical biochemical and biomechanical arrangements found in cartilaginous tissue, but also the nanotopography found in cartilaginous tissue,^6,65,66^ the effective integration of chondrocytes within the PCL + GEL fibers established here suggest that this alternated approach is a viable option for cell inclusion into 3D electrospun scaffolds during their electrospinning, and the ultimate development of highly cellularized constructs for cartilage TE.

The adoption of an immortalized cell line C28/I2 was crucial in this case not only to analyze the influence of the multiple electrospraying operational parameters, but also to do so efficiently and reproducibly.^52,67^ It should be noted, however, that these cells may be less sensitive to the process than primary chondrocytes, which is why electrospraying studies with primary chondrocytes were conducted under optimum electrospraying parameters to validate the use of this technique for cartilage TE applications. According to the data, bio-electrospraying had a slight detrimental effect on the viability of primary chondrocytes. Numerous investigations reached similar outcomes, most notably those that utilized primary cells.^15,39,40,50,68^ While primary chondrocytes are known to reproduce the metabolism and behavior of native articular cartilage more closely, they have a restricted capacity for self-renewal and repair and are more sensitive to external stimuli.^52^ Nonetheless, it should be emphasized that the viability of post-electrosprayed primary chondrocytes remained high, exceeding 70%. Despite these encouraging results, additional in vitro studies, particularly on the viability (live/dead), morphology, proliferation, and gene expression of post-electrosprayed primary chondrocytes, as well as the combination with polymer electrospinning for in-depth validation of cell state after electrospraying and electrospraying/electrospinning, are required to ultimately validate this technology for cartilage TE applications.

## Conclusions

Here, an alternative seeding methodology has been proposed and evaluated to assess its possibility for cartilage TE. Bio-electrospraying proved to be non-detrimental to C28/I2 chondrocytes under certain operational conditions. This includes an intermediate NG (27G), not only to prevent cell shearing from a smaller NG, but also to avert the solicitation of higher voltages to establish a stable cone-jet mode from the higher NG; a lower applied voltage (9 kV), since higher voltages can induce electrical and thermal damage to the cells; a smaller NCD (5 cm), to prevent cell death and increase the number of recovered cells; and an intermediate FR (2–5 mL/h) to prevent not only the shear stress on the cells of a higher FR, but also to reduce the electrospraying duration and, consequently, prolonged exposure to the electric field and lower temperatures and CO_2_ concentrations. C28/I2 chondrocyte gene expression remained unchanged after electrospraying. The long-term proliferation studies revealed that chondrocyte proliferative ability was not affected, regardless of the operational conditions. Preliminary data of an alternated approach combining chondrocyte electrospraying and polymer electrospinning showed that chondrocyte integration was possible and that the proliferative behavior of the embedded chondrocytes was retained. Besides the immortalized C28/I2 cell line, primary chondrocytes were also electrosprayed employing the optimal electrospraying parameters, revealing the higher sensitivity degree of these cells. Still, their post-electrosprayed viability remained considerably high, suggesting bio-electrospraying might be a promising alternative to the existent cell seeding techniques, promoting not only cells safe delivery to the scaffold, but also the development of cellularized cartilage tissue constructs.

## Supplemental Material

sj-docx-1-tej-10.1177_20417314211069342 – Supplemental material for Bio-electrospraying assessment toward in situ chondrocyte-laden electrospun scaffold fabrication Click here for additional data file. Supplemental material, sj-docx-1-tej-10.1177_20417314211069342 for Bio-electrospraying assessment toward in situ chondrocyte-laden electrospun scaffold fabrication by Ângela Semitela, Gonçalo Ramalho, Ana Capitão, Cátia Sousa, Alexandrina F Mendes, Paula AAP Marques and António Completo in Journal of Tissue Engineering
