# Supplementary material for: Bio-electrospraying assessment toward in situ chondrocyte-laden electrospun scaffold fabrication
Source: J Tissue Eng. 2022 Jan 8;13:20417314211069342. doi: 10.1177/20417314211069342 (PMC8743920; doi:10.1177/20417314211069342)
Supplement: sj-docx-1-tej-10.1177_20417314211069342 – Supplemental material for Bio-electrospraying assessment toward in situ chondrocyte-laden electrospun scaffold fabrication [file sj-docx-1-tej-10.1177_20417314211069342.docx]

**Bio-electrospraying assessment towards *in situ* chondrocyte-laden electrospun scaffold fabrication**

**Authors**

Ângela Semitela^1^, Gonçalo Ramalho^1^, Ana Capitão^2^, Cátia Sousa^2^, Alexandrina Mendes^2^, António Completo^1*^ and Paula A.A.P. Marques^1*^

**Affiliations**

^1^ Centre of Mechanical Technology and Automation (TEMA), Department of Mechanical Engineering, University of Aveiro, 3810-193 Aveiro, Portugal

^2^ Centre for Neuroscience and Cell Biology (CNC), University of Coimbra, Portugal

***Corresponding Authors:**

Paula A.A.P. Marques, E-mail: [paulam@ua.pt](mailto:paulam@ua.pt)

António Completo, E-mail: [completo@ua.pt](mailto:completo@ua.pt)

**SUPPLEMENTARY INFORMATION**

**Table S1.** Real time primer conditions.

| **Gene** | **Primer name** | **Primer sequence** | **Direction** | **Temp.** | **Efficiency** | **Fragment Size** |
| --- | --- | --- | --- | --- | --- | --- |
| *COL2A1* | hsa-COL2A1-RT_F | CCAGATTGAGAGCATCCGCA | Forward | 62ºC | 96% | 193 |
|  | hsa-COL2A1-RT_R | TGGATTGGGGTAGACGCAAG | Reverse |  |  |  |
| *COL1A1* | hsa-COL1A1-RT_F | TCGAGGGCCAAGACGAAGAC | Forward | 62ºC | 104% | 136 |
|  | hsa-COL1A1-RT_R | ACGTCATCGCACAACACCTTG | Reverse |  |  |  |
| *ACAN* | hsa-ACAN-RT_F | CCGAGGAGCAGGAGTTTGTC | Forward | 62ºC | 104% | 145 |
|  | hsa-ACAN-RT_R | AAGTTGTCAGGCTGGTTGGG | Reverse |  |  |  |
| *HPRT1* | hsa-HPRT1_F | TGACACTGGCAAAACAAT | Forward | 60ºC | 99% | 117 |
|  | hsa-HPRT1_F | GGCTTATATCCAACACTTCG | Reverse |  |  |  |


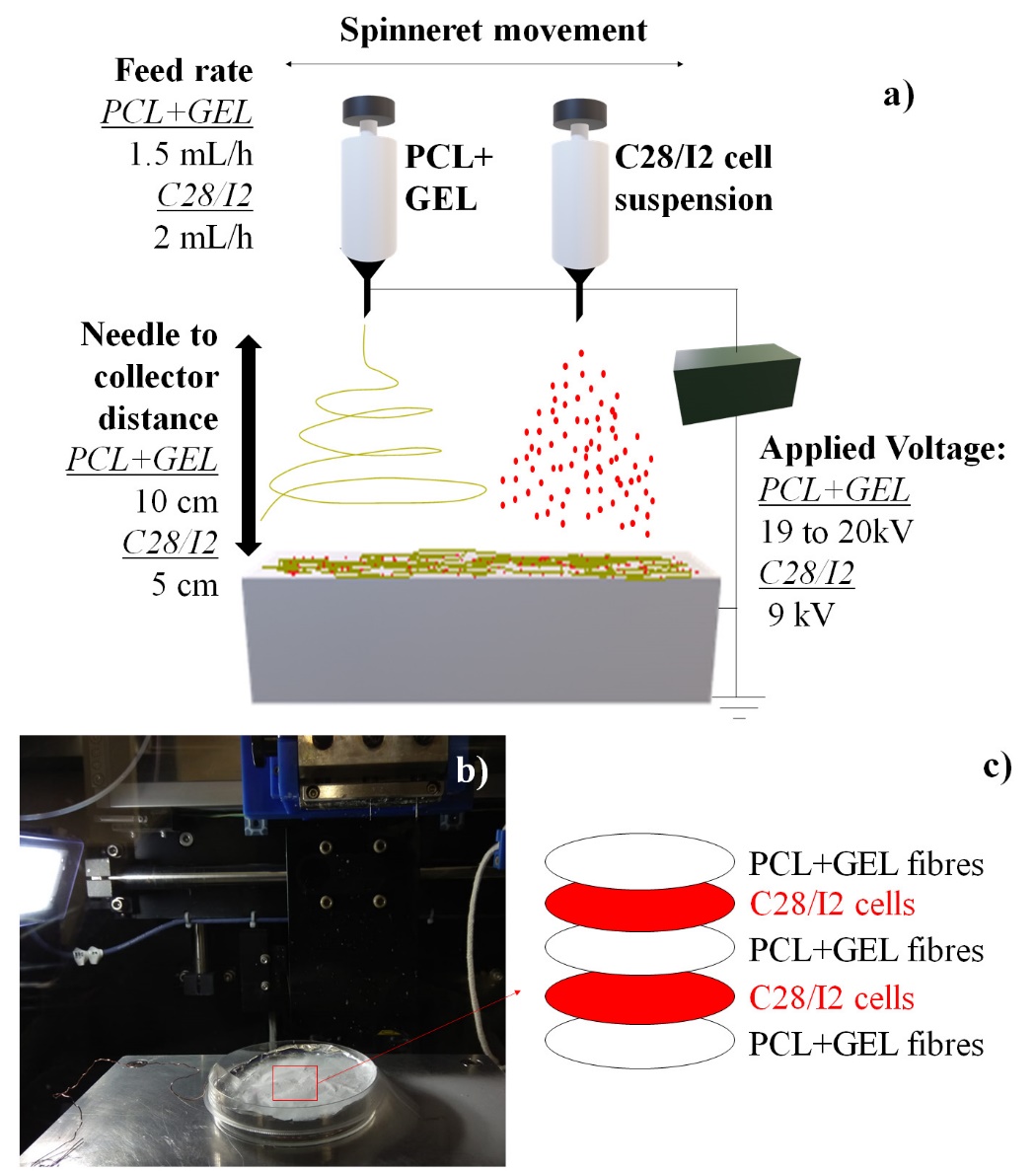


**Figure S1.** Illustrative diagram of the electrospinning and electrospraying set-up used for the alternated chondrocyte electrospraying and PCL+GEL electrospinning (a); actual experimental set-up (b) and schematic representation of the five-layer scaffold (c).

A dead control was also performed to ensure that calcein-AM and propidium iodide were working by inducing cytotoxicity with ethanol before the staining. The resulting live/dead staining image, shown in Figure S1, displays the dead adhered chondrocytes. The quantification of the live/dead covered area revealed that, while there was a very small portion of live cells (1.50 ± 0.50%), most chondrocytes were dead (32.18 ± 2.68 %).

**Figure S2.** Quantification of the area covered by live and dead cells after 24 hours on culture control (CC), needle controls (NC) using 25, 27 and 30G needles and dead control and live/dead staining image of the dead control (green corresponds to live cells and red to dead cells). Scale bar: 100 µm. Statistical analysis by One-way ANOVA followed by post hoc Tukey’s test: **p <* 0.05.


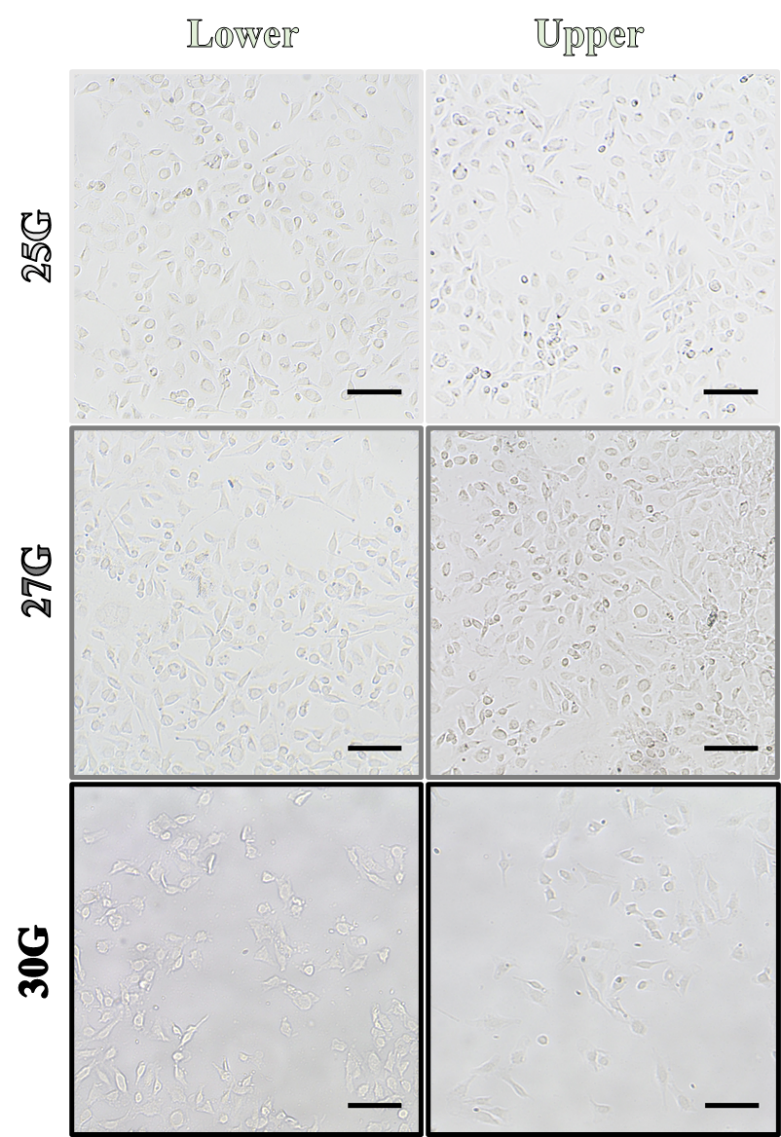


**Figure S3.** Micrographs of the post-electrosprayed chondrocytes after 24 hours after electrospraying using lower and upper volage range within the stable cone-jet mode. Scale bars: 100 µm.

**Figure S4.** Quantification of the area covered by live and dead cells after 24 hours of electrospraying using different needles gauges and needle to collector distance (NCD). Statistical analysis by One-way ANOVA followed by post hoc Tukey’s test: ^###^*p <* 0.001, ****p <* 0.001, where * denotes statistical significant differences between different NCD for each needle diameter, while # denotes statistical significant differences between different needle diameters for each NCD.


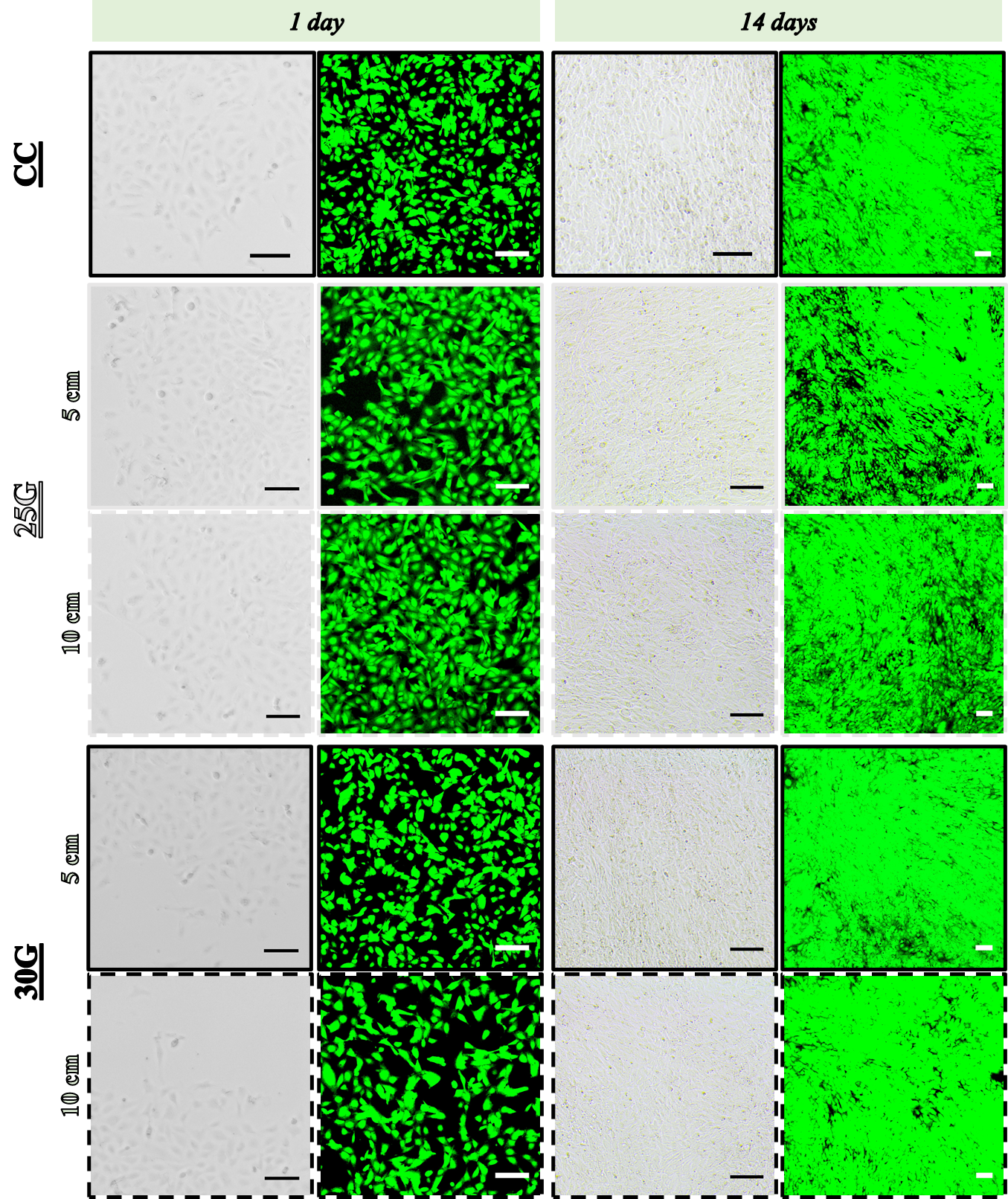


**Figure S5.** Micrographs and live/dead staining images of the post-electrosprayed chondrocytes **(**2**×**10^4^ electrosprayed C28/I2 chondrocytes were exposed to each condition combination of needle diameter and needle to collector distance.**)**, through 25 and 30G needles after 1 and 14 days of culture and the respective culture control. Green corresponds to live cells and red to dead cells. Scale bars: 100 µm.

After 14 days of culture, significant differences were observed on the viability of the post-electrosprayed chondrocytes, regardless of the NG and NCD combination (from 55.19 ± 9.77 at day 1 to 96.95 ± 3.36 % at day 14 for CC, from 49.27 ± 4.46 at day 1 to 96.91 ± 2.67 % at day 14 for 25G at 5 cm, from 50.85 ± 5.35 at day 1 to 97.67 ± 2.05 % at day 14 for 27G at 5 cm, from 47.82 ± 4.37 at day 1 to 98.36 ± 1.08 % at day 14 for 30G at 5 cm, from 44.82 ± 3.48 at day 1 to 97.11 ± 3.01 % at day 14 for 25G at 10 cm, from 45.32 ± 4.27 at day 1 to 97.61 ± 2.25 % at day 14 for 27G at 10 cm, from 47.21 ± 5.01 at day 1 to 96.68 ± 1.68 % at day 14 for 30G at 10 cm; p < 0.001).

**Figure S6.** Quantification of the area covered by live and dead cells (2**×**10^4^ electrosprayed C28/I2 chondrocytes were exposed to each condition combination of needle diameter and needle to collector distance.) after 1 and 14 days of culture using different needles gauges and needle to collector distance (NCD). Statistical analysis by by One-way ANOVA with repeated measures followed by post hoc Tukey’s test; ****p <* 0.001, where * denotes statistical significant differences different needle diameters and needle to collector distance condition combination over culture time.
